# Relation between hematocrit partitioning and red blood cell lingering in a microfluidic network

**DOI:** 10.1016/j.bpj.2024.07.042

**Published:** 2024-08-05

**Authors:** Aurelia Bucciarelli, Alberto Mantegazza, Andreas Haeberlin, Dominik Obrist

**Affiliations:** 1 ARTORG Center for Biomedical Engineering Research, University of Bern, Bern, Switzerland; 2 Department of Electronics, Information and Bioengineering, Politecnico di Milano, Milan, Italy; 3 Department of Cardiology, Bern University Hospital, University of Bern, Bern, Switzerland

## Abstract

Despite increased interest in the effect of lingering red blood cells (LRBCs) on the heterogeneous hematocrit distribution in the microcirculation, quantitative data on LRBCs before and after the lingering event are still limited. The aim of the study was to investigate the relation between red blood cell (RBC) lingering and hematocrit partitioning in a microfluidic model of a microvascular bifurcation in the limit of low hematocrit conditions (tube hematocrit <10%). To this end, the classification of LRBCs was performed based on timing, position, and velocity of the RBCs. The investigation provided statistical information on the velocity, shape, and orientation of LRBCs as well as on their lateral distribution in the parent and daughter vessels. LRBCs traveled predominantly close to the centerline of the parent vessel, but they marginated close to the distal wall in the daughter vessels. Differently than the RBC flow observed in the smallest vessels, no influence of lingering events on the local hematocrit partitioning was observed in our experiments. However, importantly, we found that LRBCs flowing in the daughter vessel after lingering may be connected to reverse hematocrit partitioning in downstream bifurcations by influencing the skewness of the hematocrit distribution in the daughter vessel, which relates to the so-called network history effect.

## Significance

Red blood cells (RBCs) play a fundamental role in oxygen transport in the microcirculation. Understanding the individual RBC dynamics at the microscale is helpful to unravel mechanisms governing the blood flow distribution in the microcirculation. In this study, the behavior of lingering RBCs (LRBCs) is investigated in a microfluidic microvascular bifurcation model. Methods for the LRBC classification are compared and information on the LRBC lateral distribution in the vessels of the bifurcation is provided. We demonstrate that LRBCs flowing in daughter vessels (after lingering) increase the hematocrit skewness, which leads to reverse partitioning in the following bifurcation. This finding highlights RBC lingering as a mechanism for hematocrit partitioning that determines the blood flow distribution in the microcirculation.

## Introduction

The microcirculation features highly interconnected networks of small capillaries ranging from 5 to 10μm in diameter (1) that are structured in a mesh-like fashion enabling efficient local mass transport of oxygen carried by red blood cells (RBCs) to the surrounding tissue. Many studies showed that the RBC distribution in microvascular networks is heterogeneous in space and time and that this heterogeneity is related to the RBC dynamics at microvascular bifurcations (2,3,4,5,6).

RBCs show a nonuniform and time-dependent distribution at divergent bifurcations (7) and preferentially enter the daughter vessel with higher blood flow rate, such that the hematocrit partitioning is higher than proportionality would suggest (8,9) (Zweifach-Fung effect). This nonlinear relationship between the fraction of blood flow and the fraction of RBC flux in the daughter vessels is known as classical hematocrit partitioning. At the same time, a reduction or an inversion of this classical partitioning was observed (10,11,12) especially in larger networks where the global RBC dynamics is more complex (5,6,13). It was reported that a reduction of the Zweifach-Fung effect is more likely to occur in the limit of low feeding hematocrit (10), for increasing inlet velocity (4,12), and for a skewed hematocrit profile (5,13).

In previous studies (5,4), we observed that an inversion of the classical hematocrit partitioning (namely, a reverse partitioning) was correlated to skewed hematocrit profiles in the parent vessels of the bifurcations with reverse partitioning. However, the fluid dynamic phenomena at the RBC scale that created these skewed hematocrit profiles remained unknown. Balogh and Bagchi (7,13) simulated a physiologically realistic microvascular network and demonstrated that competing phenomena give rise to transient events making the RBC behavior oscillate between classical and reverse partitioning. They identified the lingering of RBCs at the apex of a divergent bifurcation as one of the factors influencing the local hematocrit partitioning. Here, we will investigate whether RBC lingering at the apex of a bifurcation can also be held accountable for the skewed hematocrit profiles that were connected to reverse partitioning at the following bifurcation.

In a qualitative fashion, lingering RBCs (LRBCs) can be defined as cells that do not flow directly from the parent vessel to the daughter vessels but get stuck at the apex of a microvascular bifurcation. The LRBCs typically flow near the separation surface (i.e., the surface that separates the streamlines entering one of the daughter vessels from the streamlines that enter the other vessel). The LRBCs approach the apex with almost zero velocity, interact with the vessel wall and other RBCs, and remain at the apex for a prolonged period of time before entering one of the daughter vessels. This process is dominated by cell-cell and cell-wall interactions. It is not governed by the undisturbed streamlines that carry non-lingering RBCs (NLRBCs) and it cannot be interpreted as a simple cell deceleration with respect to the underlying plasma flow.

Although lingering has been recognized as an important phenomenon for the RBC dynamics in the microcirculation, the number of studies remains limited (13,14,15,16). As a consequence, a general consensus on a mathematical definition of lingering has yet to be achieved. It is necessary to critically evaluate and compare the different methods used to define LRBCs because the results depend strongly on the criteria used to discriminate LRBCs from NLRBCs. In previous studies, LRBCs were identified based on the minimal RBC velocity (14,15) and on the RBC residence time (13,16). In this study, we compare purely geometric and kinematic criteria (minimal distance of the RBC to the apex and the minimal RBC velocity) to the relative residence time, which can be interpreted as a combination of the geometric and kinematic criteria. We show that the latter is the most convincing method to identify LRBCs in our experimental dataset.

Previous studies (7,13,15) investigated the effect of LRBCs on the hematocrit partitioning at the same bifurcation where they linger and focused on vessels with diameters that were comparable to or smaller than the RBC size. The ratio between vessel diameter and RBC size, known as confinement ratio (*λ*), was less than unity in those experiments (i.e., high confinement). Under these conditions, LRBCs can partially block the entry of a daughter vessel, thus hindering the passage of the following RBCs approaching the bifurcation. As a result, incoming RBCs may be forced to cross the separation surface and enter the low-flow vessel favoring reverse partitioning.

In this study, we investigated whether RBC lingering can also affect the dynamics at the following bifurcation. We hypothesize that LRBCs flowing in the daughter vessel causes skewed hematocrit profiles that favor reverse partitioning at the next bifurcation, thus promoting the network history effect (5,17). To test this hypothesis and expand the current understanding of RBC lingering, we created a microfluidic device with a single symmetric diverging bifurcation to assess the local dynamics of LRBCs at conditions of low tube hematocrit. In contrast to other studies, the microchannels were bigger than the typical RBC size, resulting in a low confinement ratio (λ>1). Therefore, local blockage effects due to lingering were expected to be weaker than in high-confinement configurations.

These results from a single diverging bifurcation were then compared to data obtained from previous independent experiments with a complex network where reverse partitioning was observed (5). This comparison suggested that reverse partitioning may occur at the following bifurcation if a sufficiently high number of RBCs have lingered in the previous bifurcation.

## Materials and methods

### Device fabrication and experimental protocol

**Figure 1:**
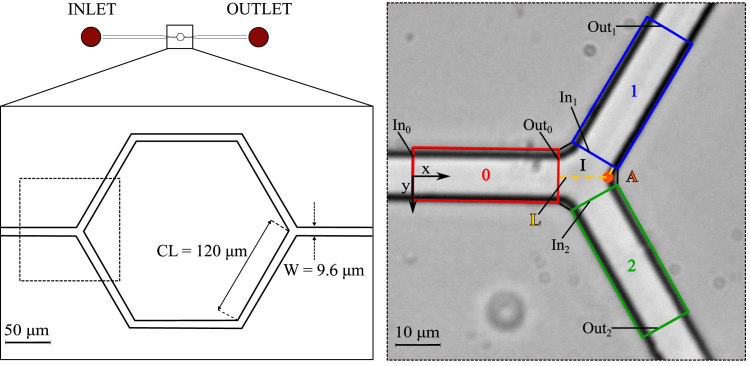
Schematic of the whole microfluidic device. Microfluidic device with inlet and outlet (*top left*), a magnified version of the single-mesh network (*bottom left*), and a microscope image (512 × 512 pixels) of the diverging bifurcation (*right*) with regions of interest (ROIs) used for the image analysis: parent vessel (ROI 0) in red, daughter vessel 1 (ROI 1) in blue, daughter vessel 2 (ROI 2) in green, and the intersection (I) in the center where the apex (*A*, *orange*) is located. The dashed yellow path of length *L* is the reference length for the definition of relative residence time $\tau_{RBC}$.

The experiments were performed using a microfluidic device made of polydimethylsiloxane and produced by conventional soft lithography and replica molding. The design used in this study was a single hexagonal loop connected upstream and downstream to wider feeding and draining microchannels, respectively (Fig. 1 left panel). The microchannels had a rectangular cross-section (width W=9.6μm, height H=8μm) and a length of CL=120μm. The diverging and converging bifurcations were symmetric with a bifurcation angle of 120°.

Fresh heparinized blood from New Zealand rabbits was provided by the Experimental Surgery Facility (University of Bern) under the animal license BE 37/19 by the veterinary authorities of the Canton of Bern. The RBCs were extracted and re-suspended in a solution with a reservoir hematocrit of $H_{r}=10\%$ following Roman et al. (18). The solution used to re-suspend the RBCs matched the viscosity of the plasma to respect the physiological viscosity contrast between the plasma and the red blood cell cytosol (19) and the density of the RBCs to prevent RBC sedimentation at the bottom of the reservoir throughout the experiments. Briefly, the suspending medium was prepared using 65% glucose-albumin-sodium-phosphate (GASP) buffer (phosphate buffer saline with 5.5 mM glucose and 4% bovine serum albumin) and 35% stock solution (90% Optiprep (Sigma-Aldrich, St. Louis, MO, USA) + 10% glucose-albumin-sodium-phosphate buffer 10 times concentrated). The RBC suspension protocol was already successively used for various blood sources (human (17,20) and porcine (4,5,12) RBCs). The plasma-like solution had the viscosity of plasma $\mu =1.96\times 10^{-3}Pa\cdot s$ at 20°*C* and the density of RBCs $\rho =1090\;kg/m^{3}$.

The experiments were carried out on an inverted microscope (Eclipse Ti-E, Nikon, Japan) with a 40× air objective (lateral resolution 0.16μm/pixel and numerical aperture 0.60) and a frame size of 512 × 512 pixels (Fig. 1, right). A perfusion pressure of Δp=213Pa was used to drive the blood flow. This generated an RBC flow with an average velocity of 0.67mm/s, which is similar to the velocities found in capillary networks at rest condition (2,21,22). At steady state, a video of 4000 frames was recorded at 395frames/s using a high-speed camera (ORCA-flash 4.0, Hamamatsu, Japan) and a minimum of 378 RBCs were tracked per video. More information on the device fabrication and experimental protocols is reported in (4,5,12).

### Image processing

The video sequence was imported and processed with custom-written Matlab scripts (MathWorks, Natick, MA, USA). For reference, all videos are available in the Supporting Material (Video S1. Recorded video of the RBC flowing through the bifurcation for a tube hematocrit Ht,0 ≈ 5.2% (feeding hematocrit Hr = 10%) slowed down five times, at 79 frames per second (fps) (original recording at 395 fps), Video S2. Recorded video of the RBC flowing through the bifurcation for a tube hematocrit Ht,0 ≈ 6.3% (feeding hematocrit Hr = 20%) slowed down five times, at 79 fps (original recording at 395 fps), Video S3. Recorded video of the RBC flowing through the bifurcation for a tube hematocrit Ht,0 ≈ 8.7% (feeding hematocrit Hr = 30%) slowed down five times, at 79 fps (original recording at 395 fps)). The preprocessing procedure consisted of five main steps: correction for rotation and background illumination difference, background subtraction, noise removal, and binarization. In the case of overlapping RBCs, the image segmentation and shape reconstruction was done in two steps: first, a general sweep by water-shedding algorithms was performed in Fiji (ImageJ) (23). Second, a manual check and correction were performed in Matlab if needed.

Video S1. Recorded video of the RBC flowing through the bifurcation for a tube hematocrit *Ht*,0 ≈ 5.2% (feeding hematocrit *Hr* = 10%) slowed down five times, at 79 frames per second (fps) (original recording at 395 fps)

Video S2. Recorded video of the RBC flowing through the bifurcation for a tube hematocrit *Ht*,0 ≈ 6.3% (feeding hematocrit *Hr* = 20%) slowed down five times, at 79 fps (original recording at 395 fps)

Video S3. Recorded video of the RBC flowing through the bifurcation for a tube hematocrit *Ht*,0 ≈ 8.7% (feeding hematocrit *Hr* = 30%) slowed down five times, at 79 fps (original recording at 395 fps)

The resulting frames were analyzed with the open-source software PTVlab (24) for particle tracking velocimetry (PTV). The validation of the PTV algorithm for RBC tracking was performed and reconfirmed in our previous studies (4,5,12). The output data from PTV were processed in Matlab to compute the following information for each RBC at each frame: RBC centroid position in the image (mm), velocity u(mm), circularity ε (−), and orientation *β* (°). The microscope image was divided into four regions of interest (ROIs): parent vessel (ROI 0), daughter vessel 1 (ROI 1), daughter vessel 2 (ROI 2), and intersection (I) (Fig. 1, right). The ROIs 0, 1, and 2 had a length of 33.2μm and a distance of 5.5μm from the center of the intersection. The lateral position within a channel was given by the normalized coordinate $y^{*}=y/W$ ranging from −0.5 to 0.5. In the daughter vessels, we define the channel walls as proximal ($y^{*}=-0.5$) and distal ($y^{*}=0.5$) according to the flow direction. The inlets (In) and outlets (Out) were defined according to the flow direction. The bifurcation apex (A) was defined as the intersection between the distal walls of the daughter vessels. Lastly, the distance Δs between the RBC centroid and the apex A was computed. The distance Δs goes to zero as an RBC approaches the intersection and increases again as the RBC enters one of the daughter vessels.

### Classification of LRBCs

To distinguish LRBCs from NLRBCs, a relative residence time $\tau_{RBC}$ was defined as the normalized time spent by an RBC at the intersection I (Fig. 1):

(1) $$\tau_{RBC}=\frac{t_{r,RBC}}{t_{ref}}\text{,}$$

where $t_{r,RBC}$ is the residence time of a specific RBC at the intersection and $t_{ref}=L/{\bar{u}}_{Out,0}$ is the reference time an RBC needs to travel the reference length *L* (from the exit of the ROI of the parent vessel to the apex and then to the entry of the daughter vessel; Fig. 1) with a velocity equal to the mean velocity at the outlet of the parent vessel (${\bar{u}}_{Out,0}$). The choice of this reference timescale defines a convective timescale for RBC transport in the bifurcation. This definition is similar to the lingering Péclet number defined by Rashidi et al. (15), except that it is a cell-based definition instead of a statistical estimation based on the probability density function of the RBC velocity. For our experiments, an RBC was defined as a LRBC if $\tau_{RBC}>2.0$. A justification a posteriori for this choice is given in the “results” section.

### Hematocrit

The mean tube hematocrit ${\bar{H}}_{t,i}$ in ROI *i* was computed as

(2) $${\bar{H}}_{t,i}=\frac{1}{N_{frames}}\sum_{j=1}^{N_{frames}}\frac{N_{rcb,j}\times MCV_{RBC}}{V_{i}}\text{,}$$

where $N_{frames}$ is the number of frames, $N_{RBC,j}$ is the number of RBCs in ROI *i* at frame *j*, and $V_{i}$ is the microchannel volume corresponding to ROI *i*. The mean corpuscular volume of New Zealand rabbit RBCs (25,26,27) is $MCV_{RBC}=68.6\;\mu m^{3}$. The mean tube hematocrit measured in the parent vessel was ${\bar{H}}_{t,0}\approx 5.2\%$, which was in good agreement with the theoretical value of $H_{t}=5.50\%$ for a reservoir hematocrit of $H_{r}=10\%$ and a hydraulic diameter of $D_{h}=\left(2\times W\times H\right)/\left(W+H\right)=8.72\;\mu m$ predicted by Pries and Secomb (28):

(3) $$\frac{H_{t}}{H_{r}}=H_{r}+\left(1-H_{r}\right)\cdot \left(1+1.7e^{-0.415D_{h}}-0.6e^{-0.011D_{h}}\right)\text{.}$$

Two experiments with different reservoir hematocrit ($H_{r}=20\%$ and $H_{r}=30\%$) were also carried out and the measured tube hematocrit was ${\bar{H}}_{t,0}\approx 6.3\%$ and ${\bar{H}}_{t,0}\approx 8.7\%$, respectively. Results for these hematocrits were not significantly different from the experiment with ${\bar{H}}_{t,0}\approx 5.2\%$ ($H_{r}=10\%$), thus detailed data from these experiments are only reported in the Supporting Material.

### Lateral RBC distribution

The lateral distribution function $LDF_{i}\left(y^{*}\right)$ of RBCs in the ROI *i* (i.e., the hematocrit profile) was computed from the histogram of the lateral position of RBCs at the inlet and outlet of the ROI *i*. The skewness index (29) for the RBC lateral distribution in the respective ROI *i* was then calculated according to

(4) $$Sk_{i}=\left|\frac{\int_{-0.5}^{0}LDF_{i}\left(y^{*}\right)dy^{*}}{\int_{-0.5}^{0.5}LDF_{i}\left(y^{*}\right)dy^{*}}-0.5\right|\text{.}$$

For Sk=0, the RBC lateral distribution is symmetrical with respect to the microchannel centerline. For Sk=±0.5, the RBC lateral distribution is such that all RBCs are located on one side of the microchannel.

To evaluate the relation between RBC lingering and hematocrit partitioning, we compared lateral distribution functions $LDF\left(y^{*}\right)$, which we obtained in a different experimental setup in a previous study where we quantified the partitioning of RBCs in several consecutive bifurcations (5), to the present lateral distribution functions obtained in a single divergent bifurcation for LRBCs and NLRBCs ($LDF_{LRBC}$ and $LDF_{NLRBC}$, respectively). To this end, a composite lateral distribution function ($LDF_{C}$) was defined as

(5) $$LDF_{C}\left(y^{*},\gamma \right)=\gamma \cdot LDF_{LRBC}\left(y^{*}\right)+\left(1-\gamma \right)\cdot LDF_{NLRBC}\left(y^{*}\right)\text{,}$$

where $LDF_{LRBC}$ and $LDF_{NLRBC}$ were measured at the end of the daughter vessels and *γ* is a weighting factor that can be interpreted as the percentage of LRBCs (lingering frequency).

An optimal lingering frequency $\hat{\gamma }$ was determined by minimizing the error E(γ) between the the composite lateral distribution $LDF_{C}$ and the previously reported lateral distribution $LDF\left(y^{*}\right)$:

(6) $$E\left(\hat{\gamma }\right)=\min_{\gamma }\;E\left(\gamma \right)\text{,}$$

(7) $$E\left(\gamma \right)=\int_{0}^{0.5}{\left[LDF\left(y^{*}\right)-LDF_{C}\left(y^{*},\gamma \right)\right]}^{2}dy^{*}\text{.}$$

Note that the squared error is only integrated over the interval [0,0.5]. This is due to the network topology used by Mantegazza et al. (5), which will be further explained in the results section.

### Blood flow rate and RBC flux

The blood flow rate was calculated from the spatial and temporal mean of the RBC velocities ${\bar{u}}_{RBC,i}$ in the ROI *i* as (4,5):

(8) $$Q_{blood,i}={\bar{u}}_{Blood,i}\times \left(W\times H\right)=\chi \times {\bar{u}}_{RBC,i}\times \left(W\times H\right)\text{,}$$

where *χ* is the coefficient accounting for the velocity difference between plasma and RBCs. Due to the Fåhraeus effect (30), the RBC velocity is typically higher than the plasma velocity. Following Sherwood et al. (29), we assumed χ=1, implying that the mean RBC velocity is equal to the mean whole-blood velocity. We show later that the mean RBC velocity in the daughter vessels is biased by LRBCs, which leads to an underestimation of the blood flow rate. Similarly, the RBC flux in the different ROI was computed as

(9) $$Q_{RBC,i}={\bar{u}}_{RBC,i}\times {\bar{H}}_{t,i}\times \left(W\times H\right)\text{.}$$

The fluxes ${\hat{Q}}_{blood,i}$ and ${\hat{Q}}_{RBC,i}$ were corrected according to satisfy mass conservation at the bifurcation. To this end, we used the procedure reported in our previous work (5) and originally developed by Pries et al. (31).

The fractional blood flow rate $\Phi_{i}$ and fractional RBC flux $\Psi_{i}$ in the daughter vessel *i* was calculated as

(10) $$\Phi_{i}=\frac{{\hat{Q}}_{blood,i}}{{\hat{Q}}_{blood,0}}\;\text{and}\;\Psi_{i}=\frac{{\hat{Q}}_{RBC,i}}{{\hat{Q}}_{RBC,0}}\text{.}$$

### Statistical analysis

For the statistical analysis of the RBC position, velocity, circularity, and orientation, we used a two-sample Kolmogorov-Smirnov test (K-S test). The K-S test was selected due to its higher sensitivity to differences in distribution shape rather than the distribution median. For the statistical analysis of $\hat{\gamma }$, we used a Mann-Whitney U test, which is primarily sensitive to differences in the distribution median. All statistical tests were performed with a significance level of p=0.05.

## Results

### Classification of NLRBCs and LRBCs

**Figure 2:**
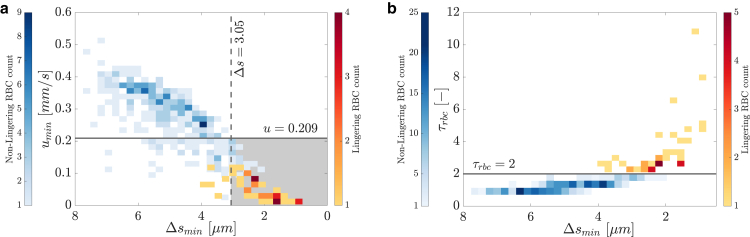
Classification of NLRBC and LRBC. (*a*) Binscatter plot of the minimal velocity in the intersection $u_{\min}$ as function of the minimal distance to the apex Δs. The solid line refers to the local minimum in the PDF of all velocities in the intersection. The dashed line refers to the radius of a rabbit RBC. The gray shaded area represents the location where RBC would be considered lingering following the method of Rashidi et al. (15). (*b*) Binscatter plot of the reference time constant $\tau_{RBC}$ as function of the minimal distance to the apex $\Delta s_{\min}$. The solid line refers to $\tau_{RBC}=2.0$.

A total of 378 RBCs were tracked throughout the experiment with ${\bar{H}}_{t,0}\approx 5.2\%$ ($H_{r}=10\%$). The minimum distance to the apex ($\Delta s_{\min}$) varied from 7.67μm to 0.86μm, whereas the minimum velocity in the intersection ($u_{\min}$) varied from 0.52mm/s to 0.004mm/s (Fig. 2 *a*).

In general, we found that the minimum RBC velocity was lower for RBCs that were closer to the apex (Fig. 2 *a*). At the same time, we also observed some very slow RBCs far away from the apex of the bifurcation. These outliers were individually checked and turned out to be RBCs that changed their shape between two consecutive frames without sensibly advancing their centroid position, such that their velocity was very low. If only $u_{\min}$ was used to distinguish LRBCs from NLRBCs, those outliers would be classified as LRBCs (i.e., false positives).

If we considered only the minimum distance $\Delta s_{\min}$ for the RBC classification, some RBCs would be falsely classified as LRBCs as they may pass very close to the apex without lingering and enter directly one of the daughter vessels. Therefore, neither the minimum distance to the apex $\Delta s_{\min}$ nor the minimum velocity at the intersection $u_{\min}$ should be used alone to determine whether an RBC lingers.

Two other methods are used by other researchers to define LRBCs, which are based on a combination of distance to apex and velocity. The first method, used by Rashidi et al. (15), defines a LRBC if the RBC is within a distance $\Delta s<r_{rbc}$ from the apex and has a velocity lower than the local minimum detected in the probability density function (PDF) of a collective dataset of RBC velocities in the mother vessel and bifurcation area. The second method, used by Pskowski et al. (16), calculates the relative residence time of each RBC, which is the normalized time spent by an RBC at the intersection, and defines LRBC as the RBCs with higher relative residence time.

We took inspiration from Pskowski et al. (16) and used the relative residence time $\tau_{RBC}$ to classify LRBCs and NLRBCs. In our experiments, we observed that $\tau_{RBC}$ varied from 0.7 to 11.0 and increased strongly when $\Delta s_{\min}$ decreased (Fig. 2 *b*), which is an illustration of the temporal heterogeneity of RBC flow at the microscale and of the variability of RBC behavior at bifurcations.

To find an appropriate threshold for the relative residence time beyond which an RBC is considered to be lingering, we compared the lingering frequency (number of LRBCs divided by the total number of RBCs) to *in vivo* observations by Kihm et al. (14), who reported lingering frequencies between 0.1 and 0.2 for a capillary bifurcation with a blood flow fraction of $0.45<\Phi_{1,2}<0.55$. For a threshold of $\tau_{RBC}=2.0$, we obtained a lingering frequency of 0.11, whereas the blood flow fraction was $\Phi_{1,2}\approx 0.5$. This is also consistent with the results of the only other in vitro study on RBC lingering that reported a lingering frequency of 0.1−0.15 for $\Phi_{1,2}=0.5$ and a reservoir hematocrit of $H_{r}<20\%$ (16). Therefore, we fixed the threshold to $\tau_{RBC}=2.0$, which resulted in 43 LRBCs and 335 NLRBCs in our experiments.

A sensitivity analysis was performed to evaluate the impact of the threshold for $\tau_{RBC}$ on the major findings of the study. Lingering frequencies of 0.18 and 0.09 were obtained for thresholds of $\tau_{RBC}=1.7$ and 2.3, respectively. Despite this change in the lingering frequency, the major results and conclusions of the study were not affected qualitatively by the choice of the threshold (within the tested range). In the following, results are presented only for the threshold $\tau_{RBC}=2.0$.

Finally, we calculated the lingering frequency using the method proposed by Rashidi et al. (15). For our experimental setup, the thresholds were $\Delta s<r_{rbc}=3.05\;\mu m$ (26) for the distance to the apex and u<0.209mm/s, which is the local minimum in the PDF of all RBC velocities in the intersection. These two thresholds are depicted in Fig. 2 *a*. With this method the lingering frequency would increase slightly to 0.14.

### Qualitative lingering analysis

**Figure 3:**
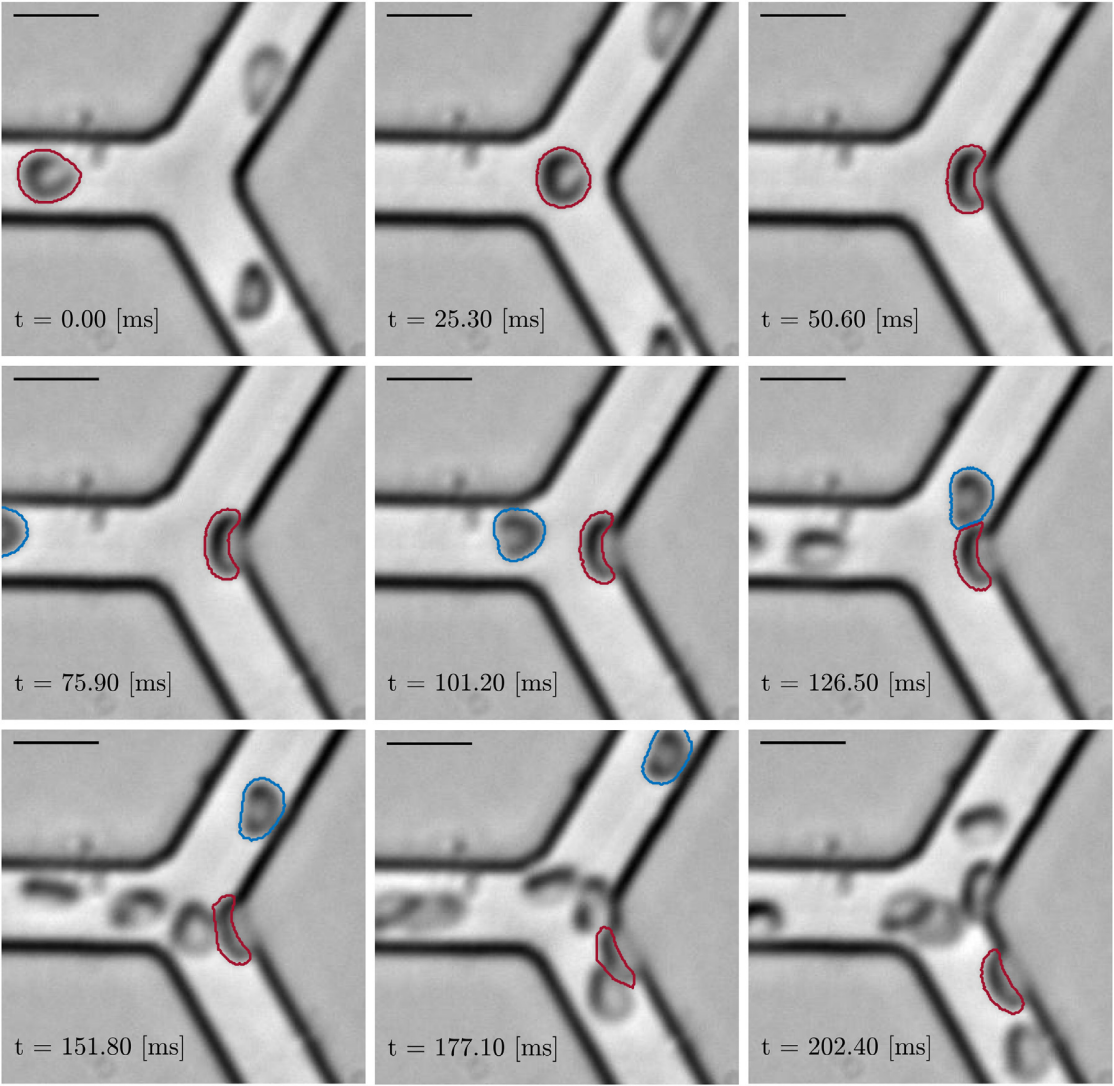
Typical lingering event. Temporal evolution of a NLRBC (*blue*) and a LRBC (*red*) approaching a divergent bifurcation. Scale bar, 10μm.

A typical lingering event is shown in Fig. 3, in which the temporal behavior and deformation dynamics are displayed for a LRBC and an NLRBC (see also the supplementary videos for dynamic illustrations of lingering events). The LRBC flows along the symmetry axis of the parent vessel while maintaining the canonical discocyte shape. At the intersection, the LRBC does not immediately enter a daughter vessel but folds around the apex of the bifurcation, partially obstructing the passage to the daughter vessels. During this process, the LRBC is subjected to a large deformation, resulting in a C-like shape. This LRBC lingers at the apex for approximately 142ms (range for all LRBCs: 45−238ms) before moving into the daughter vessel, where it leans on the distal wall of the microchannel while maintaining the deformed and elongated shape. The NLRBC also has a discoidal shape when it approaches the bifurcation but is laterally shifted to the top half of the parent vessel. The NLRBC is slightly elongated when it approaches the intersection and it takes only 18ms to cross the intersection I (i.e., five times less than the LRBC). The NLRBC has a brief cell-to-cell interaction with the lingering LRBCs, but it manages to enter the daughter vessel without appreciable deformation. This qualitative analysis of a typical lingering event shows that position, velocity, circularity, and orientation are properties that could differ between LRBCs and NLRBCs and should be investigated in more detail.

### RBC properties

In the parent vessel, LRBCs were statistically significantly faster than NLRBCs (cf. quantitative data reported in the Supporting Material) due to their concentration along the centerline. In contrast, LRBCs were significantly slower in the daughter vessels as a consequence of lingering itself. The corrected average blood flow rates (cf. Eq. 10) were ${\hat{Q}}_{blood,0}=4.76\cdot 10^{-5}\;\pm \;2.8\cdot 10^{-6}\;mm^{3}/s$, ${\hat{Q}}_{blood,1}=2.35\cdot 10^{-5}\;\pm \;2.9\cdot 10^{-6}\;mm^{3}/s$, and ${\hat{Q}}_{blood,2}=2.41\cdot 10^{-5}\;\pm \;2.2\cdot 10^{-6}\;mm^{3}/s$, resulting in average fractional blood flow rates $\Phi_{1}=0.494\;\pm \;0.046$ and $\Phi_{2}=0.506\;\pm \;0.046$. If only the mean velocity of NLRBCs is considered, the (uncorrected) blood flow rates increase to $Q_{blood,NLRBC,0}=5.16\cdot 10^{-5}mm^{3}/s$ and $Q_{blood,NLRBC,1+2}=4.91\cdot 10^{-5}mm^{3}/s$. This reduces the difference between the flow rate of the parent vessel and the sum of the flow rates of the daughter vessels by 52.1% compared to the (uncorrected) blood flow rate computed considering both the LRBC and NLRBC mean velocity. This highlights that there may be a significant error in blood flow rates determined from RBC velocities if there is a high percentage of LRBCs in the vessel of interest. In the present study, however, this error does not affect the results on LRBCs. The time-averaged fractional RBC flux in the two daughter vessels was $\Psi_{1}=\Psi_{2}=0.50$.

The corrected time-averaged fractional blood flow rates and the RBC fluxes indicate that the blood flow partitioned symmetrically in the daughter vessels, which was expected due to the symmetric geometry of the channel network. Therefore, the following results will be reported collectively without discriminating between top and bottom daughter vessel.

Further quantitative data on a series of RBC properties such as velocity, circularity, and cell orientation are reported in detail in the Supporting Material. Briefly, no difference in shape was found in the parent vessel between LRBCs and NLRBCs, which agrees well with the capillary number for the RBC flow in the parent vessel:

(11) $$Ca=\frac{\mu {\bar{u}}_{RBC,0}}{G_{s}}=\frac{1.96\cdot 10^{-3}\;Pa\;s\times 0.67\cdot 10^{-3}\;m/s}{2.5\cdot 10^{-6}\;N/m}\approx 0.55$$

where $G_{s}$ is the surface elastic shear modulus measured in optical tweezer experiments (32).

Instead, a significant shape difference was observed in the daughter vessels, where LRBCs are more elongated than NLRBCs. The LRBCs relax to a rounder shape toward the end of the daughter vessel but remain more stretched than the NLRBCs, which agrees with the previous observation that LRBCs remain close to the distal wall also at the outlet of the daughter vessels.

### Lateral RBC distribution

**Figure 4:**
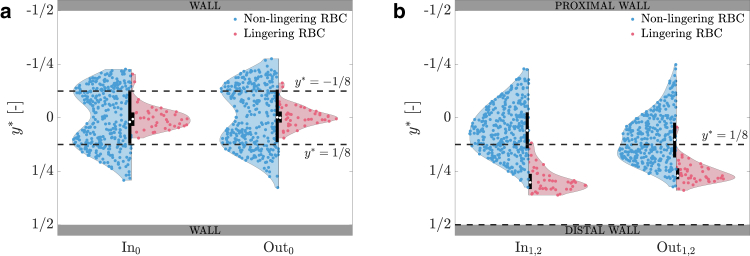
Distribution of the lateral RBC position. (*a*) Distribution of the lateral RBC position at the inlet (In_0_) and outlet (Out_0_) of the parent vessel. (*b*) Distribution of the lateral RBC position at the inlet (In_1,2_) and outlet (Out_1,2_) of the daughter vessel. Dashed lines indicate $y^{*}=\pm \frac{1}{8}$.

If we do not discriminate between NLRBCs and LRBCs, we recover the symmetric lateral RBC distribution in the parent vessel observed by Mantegazza et al. (5), where RBCs were distributed symmetrically about the center line of the parent vessel leaving a cell-depleted layer near the wall (Fig. 4 *a*, $Sk_{In,0}=0.009$ and $Sk_{Out,0}=0.01$). If we observe only NLRBCs at the inlet of the parent vessel, the lateral distribution is bimodal with cell depletion at the center of the channel such that only 52.2% of NLRBCs were found in the interval $-\frac{1}{8}<y^{*}<\frac{1}{8}$. In contrast, LRBCs are distributed in an unimodal fashion and the majority of LRBCs (95.4%) are located close to the centerline of the microchannel ($-\frac{1}{8}<y^{*}<\frac{1}{8}$). At the outlet of the parent vessel, the lateral distribution of RBCs was consistent with the distribution at the inlet: 51.9% NLRBCs and 95.4% LRBCs were located at $-\frac{1}{8}<y^{*}<\frac{1}{8}$. A two-sample K-S test indicated that the lateral distributions of NLRBCs and LRBCs in the patent vessel come from statistically different continuous distributions ($p=2.5\cdot 10^{-5}$ at In_0_, $p=6.8\cdot 10^{-5}$ at Out_0_). These results indicate that RBCs experience lingering almost exclusively if they are flowing close to the centerline of the microchannel.

In contrast, the lateral distributions of RBCs in the daughter vessels is not symmetric with respect to the centerline (Fig. 4 *b*) but skewed toward the distal wall ($Sk_{In,1,2}=0.21$, $Sk_{Out,1,2}=0.32$). The distributions for the lateral position of LRBCs and NLRBCs are both unimodal. At the entrance of the daughter vessel, the peak of the NLRBC distribution is slightly shifted to the distal wall (only 30.5% of NLRBCs are located at $\frac{1}{8}<y^{*}<\frac{1}{2}$ and $Sk_{NLRBC,In,1,2}=0.17$). In sharp contrast, 97.7% of LRBCs were found near the distal wall ($Sk_{LRBC,In,1,2}=0.50$). At the outlet of the daughter vessels, the distributions presented a similar trend: 97.7% of LRBCs and 46.0% of NLRBCs were found at $\frac{1}{8}<y^{*}<\frac{1}{2}$ ($Sk_{LRBC,Out,1,2}=0.50$ and $Sk_{NLRBC,Out,1,2}=0.30$). The K-S test confirmed that the NLRBC and LRBC lateral distributions are significantly different ($p=3.5\cdot 10^{-24}$ at In_1,2_, $p=1.1\cdot 10^{-19}$ at Out_1,2_).

### Correlation between RBC lingering and downstream reverse partitioning

**Figure 5:**
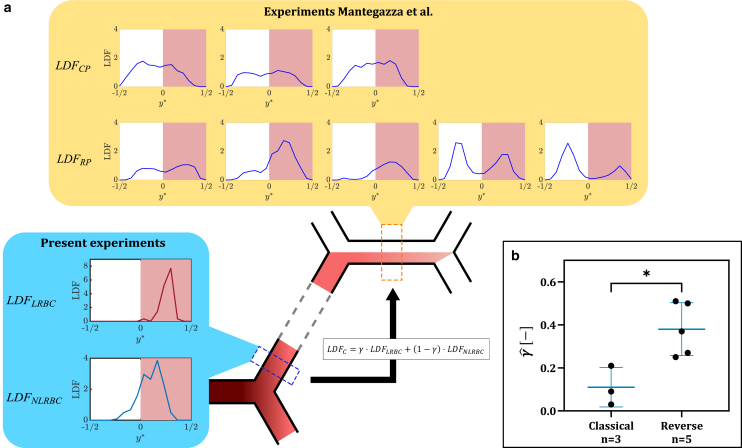
Comparison of lateral RBC distributions and optimal lingering frequencies. (*a*) Comparison of lateral RBC distributions from Mantegazza et al. (5) ($LDF_{RP,CP}$) measured in the parent vessel of four consecutive diverging bifurcations (*orange dashed box*) with the composite lateral distribution $LDF_{C}$ built from the distributions for lingering and NLRBCs ($LDF_{LRBC,NLRBC}$) measured in the daughter vessel after a diverging bifurcation (*blue dashed box*). (*b*) Optimal lingering frequencies classified for classical and reverse partitioning (∗*p* < 0.05, thin lines indicate the 25th and 75th percentiles and thick lines indicate the median values).

In Mantegazza et al. (5), eight lateral RBC distribution $LDF\left(y^{*}\right)$ are were presented. Five of these distributions were connected to reverse partitioning at the following bifurcation and the other three to classical partitioning; they are re-plotted in Fig. 5 *a* and are labeled with $LDF_{CP}$ (classical partitioning) and $LDF_{RP}$ (reverse partitioning). It was shown in that study that the skewness of the lateral distributions in the parent vessel was statistically significantly higher for reverse partitioning (mean skewness $\bar{S}k=0.21\pm 0.1$) than for classical partitioning ($\bar{Sk}=0.11\pm 0.1$).

In the present study, we observed that the presence of LRBCs in the daughter vessel after lingering can lead to strongly skewed lateral distributions in the daughter vessels, whereas NLRBCs cause much less skewing. To understand whether this effect could explain the skewed distributions observed in Mantegazza et al. (5), the composite lateral distribution function $LDF_{C}\left(y^{*},\gamma \right)$, Eq. 5, was built from a linear combination of the lateral distributions $LDF_{LRBC}$ and $LDF_{NLRBC}$ (Fig. 5 *a*) measured at the end of the daughter vessels for lingering and NLRBCs, respectively. The weighting factor *γ* can be interpreted as the lingering frequency.

Fig. 5*b* shows the optimal lingering frequencies $\hat{\gamma }$ that yielded the best fit for the composite distribution $LDF_{C}$ to the respective lateral distributions from Mantegazza et al. (5). Note that this fitting was only done in the interval $y^{*}=\left[0,0.5\right]$ (see shaded areas in Fig. 5 *a*), because the data in the interval $y^{*}=\left[-0.5,0\right]$ of $LDF_{RP}$ and $LDF_{CP}$ were dominated by inflow from another vessel. The optimal lingering frequencies $\hat{\gamma }$ vary between 0.03 and 0.51, and it appears evident that classical partitioning can be achieved only if a sufficiently small number of LRBCs flow in the daughter vessel. In contrast, a high percentage of LRBCs flowing in the daughter vessel is needed to sufficiently skew the hematocrit profile and, thus, to enable reverse partitioning in the following bifurcation. A Mann-Whitney U test indicated that $\hat{\gamma }$ for classical and reverse partitioning are statistically different ($p=3.6\cdot 10^{-2}$).

### RBC migration across the separation surface

To assess whether LRBCs also influence the hematocrit partitioning at the local bifurcation, it was tested whether RBCs departed from their original streamlines and crossed the separation surface before the intersection (i.e., whether they crossed the symmetry axis (SA) of the parent vessel). We found that only 4.8% of all RBCs crossed the SA (Table 1). For NLRBCs, this percentage (1.2%) did not differ considerably. If only LRBCs were considered, the frequency of SA crossing increased to 32.6%. This may be explained by the fact that LRBCs were more likely to flow in the center of the microchannel such that even a small interaction with a neighboring RBC can push or pull them across the SA.

**Table 1:** Percentage of RBCs Crossing the SA of the Parent Vessel for Different Groups of RBCs

All RBC	4.8%
LRBC	32.6%
NLRBC	1.2%
NLRBC without lingering event	1.2%
NLRBC during lingering event	1.2%

To further investigate whether lingering events influence the RBC distribution in the daughter vessels, the group of NLRBCs was divided into two subgroups: NLRBCs that entered the intersection while there is no lingering event, and NLRBCs that entered the intersection during a lingering event. We found 1.2% SA crossing for NLRBCs without lingering event and also 1.2% SA crossing for NLRBCs during lingering events. This indicates that lingering events had no effect on NLRBC partitioning at the local bifurcation.

## Discussion

In the present study, we studied RBCs flowing through an in vitro model of a microvascular bifurcation to understand whether LRBCs flowing in the daughter vessel after lingering may be accountable for skewing the hematocrit profiles after the local bifurcation, which may lead to reverse partitioning in the following bifurcation. Despite the measured low tube hematocrit (${\bar{H}}_{t,0}\approx 5.2\%$), the findings are physiologically relevant. The tube hematocrit falls within physiologically observed ranges (33,34,35,36). Moreover, the simulation results by Balogh and Bagchi (7) were confirmed by our previous in vitro experiments (4,5), which used similar tube hematocrit to this study, thereby reinforcing the validity and physiological relevance of our findings.

### Classification of NLRBCs and LRBCs

Four criteria for the identification of LRBCs were analyzed: minimal velocity ($u_{\min}$), minimal distance to the apex ($\Delta s_{\min}$), a combination of a threshold for velocity ($u_{\min}$) and distance to the apex (Δs), and relative residence time at the intersection ($\tau_{RBC}$). Using only the minimal distance to the apex or minimal velocity does not effectively classify RBCs (cf. Fig. 2 *a*). Combining these two factors improves the classification accuracy but still lacks the definition of a reference timescale for the RBC lingering. Fig. 2 *a* (gray area) shows that using these thresholds alone increases the number of LRBCs but misses RBCs that linger on top of other LRBCs slightly farther from the apex. These LRBCs spend a long time in the intersection and, therefore, affect incoming RBCs.

The criterion based on the relative residence time at the intersection yielded the best classification of RBCs. This criterion combines minimal velocity (LRBCs are RBCs stuck at the bifurcation with very low velocity, leading to a longer residence time in the intersection) and distance to the apex (LRBCs have a small minimal distance from the apex, requiring them to travel a longer path through the intersection, which also results in a longer residence time). Additionally, it considers the total amount of time spent in the intersection. A LRBC is not just a slow RBC near the apex; it is an RBC that remains stuck in the intersection for an extended period of time. The relative residence time criterion addresses the definition of a reference timescale for the lingering, which is overlooked by only considering a combination of thresholds for velocity and distance to the apex.

The threshold for lingering was set to $\tau_{RBC}=2.0$, which resulted in a lingering frequency of 0.11. This is consistent with *in vivo* experimental data by Kihm et al. (14), who reported a lingering frequency of 0.1−0.2. In the only other in vitro study on RBC lingering, Pskowski et al. (16) measured a lingering frequency of 0.10−0.15 for 2% and 20% feeding hematocrit, which agrees well with our experimental findings. This study also used a relative residence time criterion to identify LRBCs ($\tau_{RBC}>1.25$). However, they did not indicate whether the residence time was measured only in the bifurcation region or whether it was the whole time from the inlet to the outlet of the microfluidic device. We opted to measure the residence time only at the bifurcation because it is the region where lingering happens. We suspect that the different thresholds for $\tau_{RBC}$ between the present study and Pskowski et al. (16) are simply a consequence of different ROIs chosen for the data analysis.

### Lingering probability

The lingering probability is strongly related to the lateral position of RBCs in the parent vessel. LRBCs were concentrated around the centerline of the parent vessel, whereas only few NLRBCs were found in that region. The RBCs close to the centerline ($-\frac{1}{8}<y^{*}<\frac{1}{8}$) had a lingering probability of 19.0%, whereas this probability dropped to 1.8% for more marginated RBCs. Additionally, the majority of all lingering events (76.9%) occur during phases where the tube hematocrit is higher than the mean tube hematocrit. These results indicate that the low capillary number in the present experiments (Ca≈0.55) does not prevent lingering at the bifurcation because the development of lingering mainly depends on cell-cell and cell-wall interactions. For RBCs flowing along the centerline of the parent vessel, the elastic forces developed by the RBC membrane dominate on the viscous forces from the fluid due to the low capillary number. Nevertheless, they are in any case more likely to be LRBCs due to the prolonged stagnation that they experience at bifurcations when they interact with the apex. As a result, they need more time than the RBCs flowing close to the parent vessel walls to recover their shape and enter one of the daughter branches.

### Effect of lingering on hematocrit partitioning

At the local bifurcation, no effect of RBC lingering on hematocrit partitioning was observed. This is in contrast to the findings of Balogh and Bagchi (7), who showed that cell-to-cell and cell-wall interactions at the bifurcation influence the RBC distribution. Barber et al. (37) showed that various types of cell-to-cell interaction exist at a diverging bifurcation, namely trade-off, herding, and following interactions. The trade-off interaction, where the trailing RBC enters the opposite branch to the leading RBC, occurs most frequently for an equal flow rate split between the daughter branches, which is the case for the present experiments. Similarly to what we observed, the trade-off interaction results in a more uniform hematocrit partitioning (37). Instead, Rashidi et al. (15) found that lingering may either enhance the Zweifach-Fung effect or induce reverse partitioning. We attribute these conflicting results to the low confinement in our experiments, whereas the simulations reported in Balogh and Bagchi (7) were performed for highly confined configurations such that LRBCs had a higher impact on the following incoming RBCs.

The present results show, however, that LRBCs flowing in the daughter vessel after lingering may influence the RBC distribution at the following bifurcation. We have been able to show that a higher percentage of LRBCs flowing in the daughter vessel after a bifurcation is correlated to reverse partitioning in the following bifurcation, whereas classical partitioning seems to be favored if a smaller percentage of LRBCs is present in the daughter vessel (Fig. 5 *b*). This result adds to previous findings (5,7,12), which connected reverse partitioning to low hematocrit, increased inlet velocity, and a skewed lateral distribution. Furthermore, this result is closely related to the “history effect” described by Merlo (17), which refers to the phenomenon that the history of an RBC, i.e., the RBC dynamics in previous bifurcations, affects the RBC behavior at the local bifurcation.

The connection between lingering at a previous bifurcation and the hematocrit distribution at the following bifurcation highlights that RBC transport in the microcirculation is governed by the interplay of all bifurcations in the whole capillary network, rather than just by the RBC behavior at independent bifurcations.

### Experiments at higher hematocrit

Next to the experiment with a tube hematocrit of ${\bar{H}}_{t,0}\approx 5.2\%$ ($H_{r}=10\%$), two additional experiments were performed with $H_{r}=20\%$ and $H_{r}=30\%$ to investigate the influence of local hematocrit on RBC lingering. The results of these experiments, which are reported in the Supporting Material, were in qualitative agreement with the findings for ${\bar{H}}_{t,0}\approx 5.2\%$ ($H_{r}=10\%$). The measured tube hematocrit was ${\bar{H}}_{t}=6.3\%$ and ${\bar{H}}_{t}=8.8\%$ for a reservoir hematocrit of $H_{r}=20\%$ and $H_{r}=30\%$, respectively. Therefore, the tube hematocrit was in the same range for all three experiments despite the larger difference in the feeding hematocrit. We suspect that this is the reason why we did not observe any major difference in the results as a function of the feeding hematocrit. We conclude that small differences in tube hematocrit do not have a noticeable effect on lingering. This is in line with Pskowski et al. (16), who did not find any change in lingering frequency for reservoir hematocrits of 2% and 20%.

## Conclusions

Flowing LRBCs in the daughter vessel increases the skewness of the lateral RBC distribution of the vessel. Because a skewed distribution before a bifurcation may promote reverse hematocrit partitioning, our study suggests that flowing LRBCs in the daughter vessel after lingering can be connected to reverse hematocrit partitioning in the following bifurcation. A quantitative analysis suggests that reverse partitioning in the following bifurcation occurs if a critical lingering frequency is surpassed in the previous bifurcation. At the same time, we found that lingering had no noticeable influence on the hematocrit partitioning in the local bifurcation, which is probably related to the low confinement ratio in the present experiment, which prevents LRBCs from temporarily occluding the daughter branches.

## Data and code availability

PTV was performed with PTVlab, an open-source software available on the MathWorks File Exchange and at http://ptvlab.blogspot.com/. Custom scripts for all additional analyses we performed will be available upon reasonable request by contacting the corresponding author.

## Author contributions

A.B. and A.M. contributed equally. A.B., data curation (lead), formal analysis (equal), writing – original draft (equal), writing – review & editing (equal); A.M., investigation (lead), formal analysis (equal), writing – original draft (equal), writing – review & editing (equal); A.H., formal analysis (supporting), writing – review and editing (supporting); D.O., supervision (lead), writing – original draft (supporting), writing – review & editing (lead).

## Supporting Material

Document S1. Figures S1–S8 and Tables S1–S5

Document S2. Article plus Supporting Material

## References

[1] Pappano A.J., Gil Wier W. (2013). Cardiovascular Physiology.

[2] Schulte M.L., Wood J.D., Hudetz A.G. (2003). Cortical electrical stimulation alters erythrocyte perfusion pattern in the cerebral capillary network of the rat. Brain Res..

[3] Kleinfeld D., Mitra P.P., Denk W. (1998). Fluctuations and stimulus-induced changes in blood flow observed in individual capillaries in layers 2 through 4 of rat neocortex. Proc. Natl. Acad. Sci. USA.

[4] Mantegazza A., Ungari M., Obrist D. (2020). Local vs. global blood flow modulation in artificial microvascular networks: effects on red blood cell distribution and partitioning. Front. Physiol..

[5] Mantegazza A., Clavica F., Obrist D. (2020). In vitro investigations of red blood cell phase separation in a complex microchannel network. Biomicrofluidics.

[6] Balogh P., Bagchi P. (2017). A computational approach to modeling cellular-scale blood flow in complex geometry. J. Comput. Phys..

[7] Balogh P., Bagchi P. (2018). Analysis of red blood cell partitioning at bifurcations in simulated microvascular networks. Phys. Fluids.

[8] Pries A.R., Secomb T.W., Gross J.F. (1990). Blood Flow in Microvascular Networks. Circ. Res..

[9] Fung Y.-C. (1973). Stochastic flow in capillary blood vessels. Microvasc. Res..

[10] Shen Z., Coupier G., Podgorski T. (2016). Inversion of hematocrit partition at microfluidic bifurcations. Microvasc. Res..

[11] Hyakutake T., Abe H., Tsutsumi Y. (2022). In vitro study on the partitioning of red blood cells using a microchannel network. Microvasc. Res..

[12] Clavica F., Homsy A., Obrist D. (2016). Red blood cell phase separation in symmetric and asymmetric microchannel networks: effect of capillary dilation and inflow velocity. Sci. Rep..

[13] Balogh P., Bagchi P. (2017). Direct numerical simulation of cellular-scale blood flow in 3D microvascular networks. Biophys. J..

[14] Kihm A., Quint S., Wagner C. (2021). Lingering dynamics in microvascular blood flow. Biophys. J..

[15] Rashidi Y., Simionato G., Darras A. (2023). Red blood cell lingering modulates hematocrit distribution in the microcirculation. Biophys. J..

[16] Pskowski A., Bagchi P., Zahn J.D. (2021). Investigation of red blood cell partitioning in an in vitro microvascular bifurcation. Artif. Organs.

[17] Merlo A. (2018).

[18] Roman S., Lorthois S., Risso F. (2012). Velocimetry of red blood cells in microvessels by the dual-slit method: Effect of velocity gradients. Microvasc. Res..

[19] Mantegazza A., De Marinis D., de Tullio M.D. (2024). Red blood cell transport in bounded shear flow: On the effects of cell viscoelastic properties. Comput. Methods Appl. Mech. Eng..

[20] Roman S., Merlo A., Lorthois S. (2016). Going beyond 20 μm-sized channels for studying red blood cell phase separation in microfluidic bifurcations. Biomicrofluidics.

[21] Hudetz A.G. (1997). Blood flow in the cerebral capillary network: a review emphasizing observations with intravital microscopy. Microcirculation.

[22] Schmid F., Barrett M.J.P., Jenny P. (2019). Red blood cells stabilize flow in brain microvascular networks. PLoS Comput. Biol..

[23] Schindelin J., Arganda-Carreras I., Cardona A. (2012). Fiji: an open-source platform for biological-image analysis. Nat. Methods.

[24] Brevis W., Niño Y., Jirka G.H. (2011). Integrating cross-correlation and relaxation algorithms for particle tracking velocimetry. Exp. Fluid.

[25] Kim J.-C., Yun H.-I., Chung M.-K. (2002). Haematological changes during normal pregnancy in New Zealand White rabbits: a longitudinal study. Comp. Clin. Pathol..

[26] Lewis J.H. (1996). Comparative Hemostasis in Vertebrates.

[27] Windberger U., Bartholovitsch A., Heinze G. (2003). Whole blood viscosity, plasma viscosity and erythrocyte aggregation in nine mammalian species: reference values and comparison of data. Exp. Physiol..

[28] Pries A.R., Secomb T.W. (2008). Microcirculation.

[29] Sherwood J.M., Holmes D., Balabani S. (2014). Spatial distributions of red blood cells significantly alter local haemodynamics. PLoS One.

[30] Fåhraeus R. (1929). The suspension stability of the blood. Physiol. Rev..

[31] Pries A.R., Ley K., Gaehtgens P. (1989). Red cell distribution at microvascular bifurcations. Microvasc. Res..

[32] Hénon S., Lenormand G., Gallet F. (1999). A New Determination of the Shear Modulus of the Human Erythrocyte Membrane Using Optical Tweezers. Biophys. J..

[33] Lipowsky H.H., Usami S., Chien S. (1980). In vivo measurements of “apparent viscosity” and microvessel hematocrit in the mesentery of the cat. Microvasc. Res..

[34] Desjardins C., Duling B.R. (1987). Microvessel hematocrit: measurement and implications for capillary oxygen transport. Am. J. Physiol..

[35] Pries A.R., Ley K., Gaehtgens P. (1986). Generalization of the Fahraeus principle for microvessel networks. Am. J. Physiol..

[36] Sarelius I.H., Duling B.R. (1982). Direct measurement of microvessel hematocrit, red cell flux, velocity, and transit time. Am. J. Physiol..

[37] Barber J.O., Restrepo J.M., Secomb T.W. (2011). Simulated Red Blood Cell Motion in Microvessel Bifurcations: Effects of Cell–Cell Interactions on Cell Partitioning. Cardiovasc. Eng. Technol..

