# Supplementary material for: Relation between hematocrit partitioning and red blood cell lingering in a microfluidic network
Source: Biophys J. 2024 Aug 5;123(19):3355–65. doi: 10.1016/j.bpj.2024.07.042 (PMC11480766; doi:10.1016/j.bpj.2024.07.042)
Supplement: Document S1. Figures S1–S8 and Tables S1–S5 [file mmc1.pdf]

Biophysical Journal, Volume *123*

Supplemental information

Relation between hematocrit partitioning and red blood cell lingering in a microfluidic network

Aurelia Bucciarelli, Alberto Mantegazza, Andreas Haeberlin, and Dominik Obrist

Supplementary Material: Relation between hematocrit partitioning and red blood cell lingering in a microfluidic network

A. Bucciarelli[1,*], A. Mantegazza[2,1], A. Haeberlin[3,1], and D. Obrist[1]

[1]ARTORG Center for Biomedical Engineering Research, University of Bern, 3010, Bern, Switzerland
[2]Department of Electronics, Information and Bioengineering, Politecnico di Milano, 20133, Milan, Italy
[3]Department of Cardiology, Bern University Hospital, University of Bern, Switzerland
[*]Correspondence: aurelia.bucciarelli@unibe.ch

SUPPLEMENTAL VIDEOS

The supplementary video in .avi format can be retrieved http://www.biophysj.org. Raw-quality video will be available upon reasonable request by contacting the corresponding author.

Supplemental video S1: Recorded video of the RBC flowing through the bifurcation for a tube hematocrit $\overline{H}_{t,0} \approx 5.2\%$ (feeding hematocrit $H_r = 10\%$) slowed down 5 times, at 79 frames per second (original recording at 395 fps).

Supplemental video S2: Recorded video of the RBC flowing through the bifurcation for a tube hematocrit $\overline{H}_{t,0} \approx 6.3\%$ (feeding hematocrit $H_r = 20\%$) slowed down 5 times, at 79 frames per second (original recording at 395 fps).

Supplemental video S3: Recorded video of the RBC flowing through the bifurcation for a tube hematocrit $\overline{H}_{t,0} \approx 8.7\%$ (feeding hematocrit $H_r = 30\%$) slowed down 5 times, at 79 frames per second (original recording at 395 fps).

ADDITIONAL ANALYSIS FOR EXPERIMENT WITH $H_r = 10\%$

In addition to the lateral RBC distributions presented in the main text, we report here an analysis on other properties for NLRBCs and LRBCs such as RBC velocity, circularity and orientation.

Velocity

Fig. S1a shows that LRBCs are statistically significantly faster than NLRBCs in the parent vessel (K-S test: p-value of $1.2 \cdot 10^{-4}$ at In_0 and $7.4 \cdot 10^{-3}$ at Out_0). This is related to the observation that LRBCs are mostly located close to the centerline of the channel at the inlet of the parent vessel where the flow velocity was the highest (main text, Fig. 4).

At the outlet of the daughter vessels, the NLRBC velocity remained similar to the inlet ($\tilde{u}_{NLRBC} = 0.33\,mm/s$). The LRBC velocity increased, but it remained statistically lower than the NLRBC velocity: $\tilde{u}_{LRBC} = 0.24\,mm/s$ (K-S test: $p = 4.4 \cdot 10^{-15}$ at $\text{Out}_{1,2}$). This velocity difference is probably connected to the lateral position of the LRBCs, which remained very close to the microchannel distal wall when flowing in the daughter vessels.

Circularity

As illustrated in Fig. 3 in the main text, RBCs may undergo large deformations when they flow through the microchannel, especially if they linger at the apex of the bifurcation. To quantify this phenomenon, we measured the circularity ϵ, which is a measure of the roundness of an object ($\epsilon = 1$ is a perfect circle). In the parent vessel, the circularity distribution for NLRBCs and LRBCs was unimodal with peaks at $\tilde{\epsilon}_{NLRBC,In_0} = 0.77$, $\tilde{\epsilon}_{NLRBC,Out_0} = 0.78$, $\tilde{\epsilon}_{LRBC,In_0} = 0.73$ and $\tilde{\epsilon}_{LRBC,Out_0} = 0.77$ (Fig. S2a). These distributions are statistically different at the inlet but not at the outlet of the ROI_0 ($p = 4.4 \cdot 10^{-2}$ at In_0, $p = 0.71$ at Out_0, respectively). Generally, all RBCs were highly circular and not subjected to a significant state of deformation as they approached the bifurcation. An example of a physiological discocyte is depicted in Fig. S2a ($\epsilon = 0.83$).

In the daughter vessels, NLRBCs had a broader circularity distribution than LRBCs, but the medians were similar ($\tilde{\epsilon}_{NLRBC,In_{1,2}} = 0.76$ and $\tilde{\epsilon}_{NLRBC,Out_{1,2}} = 0.78$). In contrast, LRBCs were highly deformed at the inlet of the daughter vessels

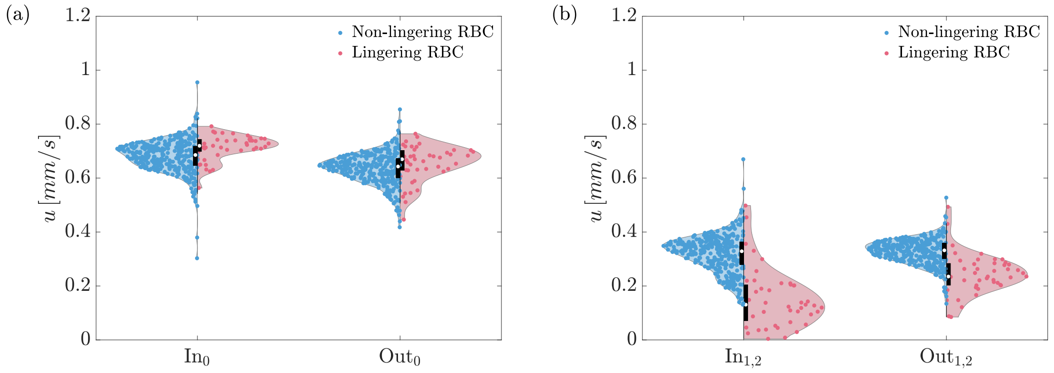

Supplemental Figure S1: RBC velocity distribution in the parent and daughter vessels. **(a)** RBC velocity at the inlet (In_0) and outlet (Out_0) of the parent vessel. **(b)** RBC velocity at the inlet ($\mathrm{In}_{1,2}$) and outlet ($\mathrm{Out}_{1,2}$) of the daughter vessel.

(Fig. S2b) with a median circularity of $\tilde{\epsilon}_{LRBC,In_{1,2}} = 0.51$. This is confirmed visually by the example in Fig. 3 in the main text, which shows a LRBC that elongates at the intersection ($\bar{\epsilon}_I = 0.63$) and holds its deformed shape when it leaves the bifurcation region. In the daughter vessels, the LRBCs partially relaxed, but they did not recover their initial discoid shape ($\tilde{\epsilon}_{LRBC,Out_{1,2}} = 0.65$). The difference in circularity distribution between NLRBCs and LRBCs in the daughter vessels is statistically significant ($p = 2.9 \cdot 10^{-21}$ at $\mathrm{In}_{1,2}$, $p = 3.9 \cdot 10^{-10}$ at $\mathrm{Out}_{1,2}$). This implies that the lingering has an influence on the shape of the RBCs after the bifurcation.

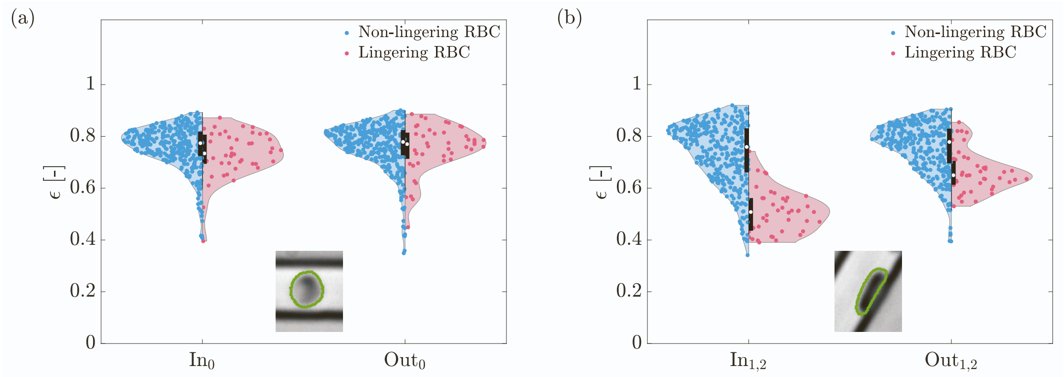

Supplemental Figure S2: RBC circularity (ϵ) distribution. $\epsilon = 1$ represents a perfect circle. **(a)** Circularity at the inlet (In_0) and outlet (Out_0) of the parent vessel. In the inset is depicted an RBC with $\epsilon = 0.83$. **(b)** Circularity at the inlet ($\mathrm{In}_{1,2}$) and outlet ($\mathrm{Out}_{1,2}$) of the daughter vessels. In the inset is depicted an RBC with $\epsilon = 0.57$.

Orientation

The orientation β was defined as the angle between the major axis of the ellipse fitted to each individual RBC and the axis of each vessel. Because the RBCs typically featured a circular discocyte shape in the parent vessel, the orientation was difficult to measure in that region. Even a minor variation in the cell aspect ratio resulted in a significant orientation change. As a result, we found that both NLRBCs and LRBCs had a broad orientation distribution in the parent vessel (Fig. S3a). It seemed that NLRBCs aligned more with the main flow direction than the LRBCs ($p = 2.0 \cdot 10^{-1}$ at In_0, $p = 9.4 \cdot 10^{-4}$ at Out_0). However, NLRBCs lost their preferential orientation at the entrance of the daughter vessel (Fig. S3b). In contrast, LRBCs were aligned with the main flow direction and just slightly tilted upwards towards the center of the vessel. This is represented by a narrow orientation distribution and is related to the fact that LBRCs in the daughter vessels were leaning on the distal wall (Fig. S2b).

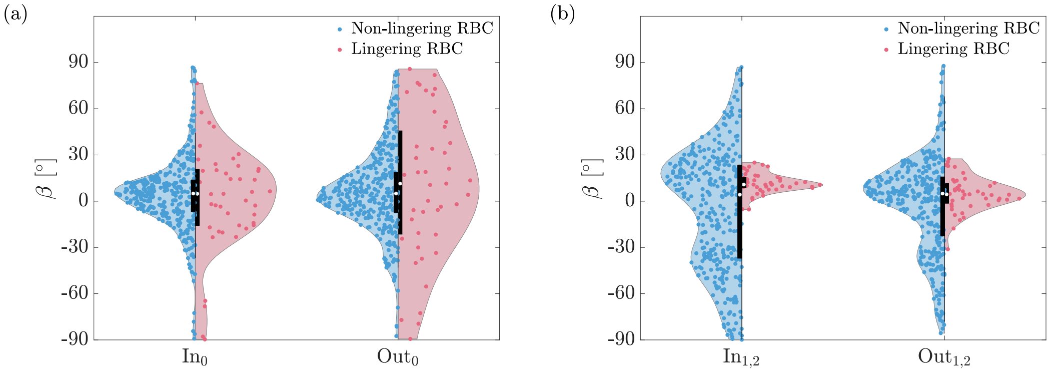

Supplemental Figure S3: RBC orientation (β) distribution. $\beta = 0°$ indicates that an RBC is aligned with the centerline of the respective ROI (i.e. the RBC follows the main flow direction). **(a)** Orientation at the inlet (In_0) and outlet (Out_0) of the parent vessel. **(b)** Orientation at the inlet ($\mathrm{In}_{1,2}$) and outlet ($\mathrm{Out}_{1,2}$) of the daughter vessels.

RESULTS FOR $H_r = 20\%$ AND $H_r = 30\%$

For the experiment at $\overline{H}_{t,0} \approx 6.3\%$ ($H_r = 20\%$) a total of 522 RBCs were tracked, thereof 50 LRBCs and 472 NLRBCs resulting in a lingering frequency of 9.6%. For the experiment at $\overline{H}_{t,0} \approx 8.7\%$ ($H_r = 30\%$) a total of 776 RBCs were tracked, thereof 80 LRBCs and 696 NLRBCs resulting in a similar lingering frequency of 10.3%.

Hematocrit lateral distribution measurements

Detailed information on the RBC hematocrit distribution (i.e. RBC lateral distribution for LRBCs and NLRBCs) are reported in Table S1. Figure S4 shows the distribution of the RBC lateral position in the parent and daughter vessels for $\overline{H}_{t,0} \approx 6.3\%$ ($H_r = 20\%$) and $\overline{H}_{t,0} \approx 8.7\%$ ($H_r = 30\%$). The shape of the lateral position distribution is similar to the results for $\overline{H}_{t,0} \approx 5.2\%$ ($H_r = 10\%$, main text, Fig. 3). This reinforces our conclusion that LRBCs are concentrated in the center of the parent vessel and that they flow near the distal wall in the daughter vessels, whereas NLRBCs are shifted to the side in the parent vessel and flow closer to the centerline of the daughters vessels.

Supplemental Table S1: Percentage of RBCs located at the lateral position $-1/8 < y^* < 1/8$ in the parent vessel (0) and $1/8 < y^* < 1/2$ in the daughter vessels (1,2) for $\overline{H}_{t,0} \approx 6.3\%$ ($H_r = 20\%$) and $\overline{H}_{t,0} \approx 8.7\%$ ($H_r = 30\%$). A two-sample Kolmogorov–Smirnov test was performed to evaluate statistical differences between the LRBC and NLRBC lateral position distributions.

	$\overline{H}_{t,0} \approx 6.3\%$ [$H_r = 20\%$]				$\overline{H}_{t,0} \approx 8.7\%$ [$H_r = 30\%$]			
	$-1/8 < y^* < 1/8$		$1/8 < y^* < 1/2$		$-1/8 < y^* < 1/8$		$1/8 < y^* < 1/2$	
	In_0	Out_0	$In_{1,2}$	$Out_{1,2}$	In_0	Out_0	$In_{1,2}$	$Out_{1,2}$
**LRBC**	88.0%	92.0%	94.0%	94.0%	85.0%	93.8%	98.8%	97.5%
**NLRBC**	52.1%	51.1%	30.7%	48.7%	50.7%	48.3%	27.9%	43.0%
p-value	$8.2{\cdot}10^{-4}$	$1.2{\cdot}10^{-5}$	$4.4{\cdot}10^{-36}$	$3.7{\cdot}10^{-21}$	$5.3{\cdot}10^{-4}$	$5.0{\cdot}10^{-6}$	$1.8{\cdot}10^{-45}$	$8.8{\cdot}10^{-29}$

Correlation between RBC lingering and downstream reverse partitioning

Similarly to the results reported for $\overline{H}_{t,0} \approx 5.2\%$ ($H_r = 10\%$), we calculated the best-fitting $\widehat{\gamma}$ for the composite lateral distribution functions measured in this study LDF_C (for $\overline{H}_{t,0} \approx 6.3\%$ [$H_r = 20\%$] and $\overline{H}_{t,0} \approx 8.7\%$ [$H_r = 30\%$]) to each lateral distribution function LDF reported by Mantegazza et al. (1). We found $0 \leq \widehat{\gamma} \leq 0.45$ and $0 \leq \widehat{\gamma} \leq 0.43$ for for $\overline{H}_{t,0} \approx 6.3\%$ ($H_r = 20\%$) and $\overline{H}_{t,0} \approx 8.7\%$ ($H_r = 30\%$), respectively. Fig. S5 shows that a statistically significantly higher percentage of LRBCs is needed to obtain a skewed hematocrit distribution that leads to reverse partitioning (M-W-U test: $p = 3.6 \cdot 10^{-2}$ and $p = 3.6 \cdot 10^{-2}$ for $\overline{H}_{t,0} \approx 6.3\%$ [$H_r = 20\%$] and $\overline{H}_{t,0} \approx 8.7\%$ [$H_r = 30\%$], respectively.)

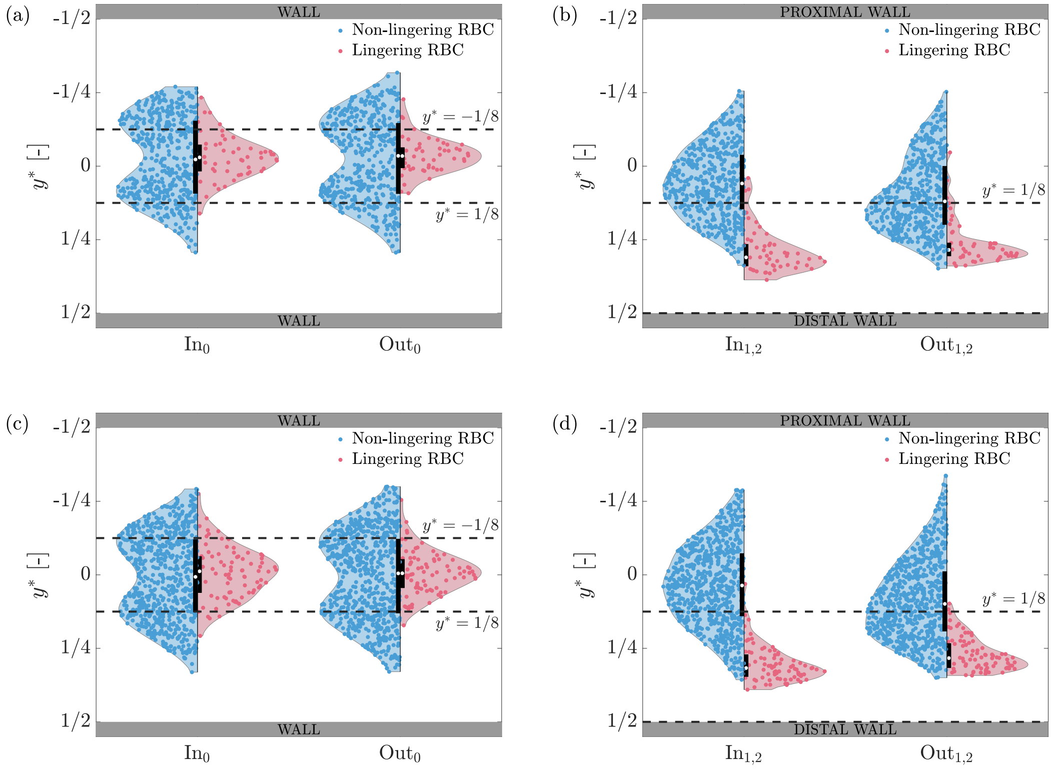

Supplemental Figure S4: Distribution of the RBC lateral position in the parent and daughter vessels for $\overline{H}_{t,0} \approx 6.3\%$ ($H_r = 20\%$) and $\overline{H}_{t,0} \approx 8.7\%$ ($H_r = 30\%$). The position of the RBC centroid is normalized with respect to the channel width ($y^* = y/W$). $y^* = 0$ is the centerline of the microchannel, whereas $y^* = 1/2$ and $y^* = -1/2$ are the distal and proximal wall of the microchannel, respectively. **(a)** Distribution of the RBC lateral position at the inlet (In$_0$) and outlet (Out$_0$) of the parent vessel for $\overline{H}_{t,0} \approx 6.3\%$ ($H_r = 20\%$). **(b)** Distribution of the RBC lateral position at the inlet (In$_{1,2}$) and outlet (Out$_{1,2}$) of the daughter vessels for $\overline{H}_{t,0} \approx 6.3\%$ ($H_r = 20\%$). **(c)** Distribution of the RBC lateral position at the inlet (In$_0$) and outlet (Out$_0$) of the parent vessel for $\overline{H}_{t,0} \approx 8.7\%$ ($H_r = 30\%$). **(d)** Distribution of the RBC lateral position at the inlet (In$_{1,2}$) and outlet (Out$_{1,2}$) of the daughter vessels for $\overline{H}_{t,0} \approx 8.7\%$ ($H_r = 30\%$).

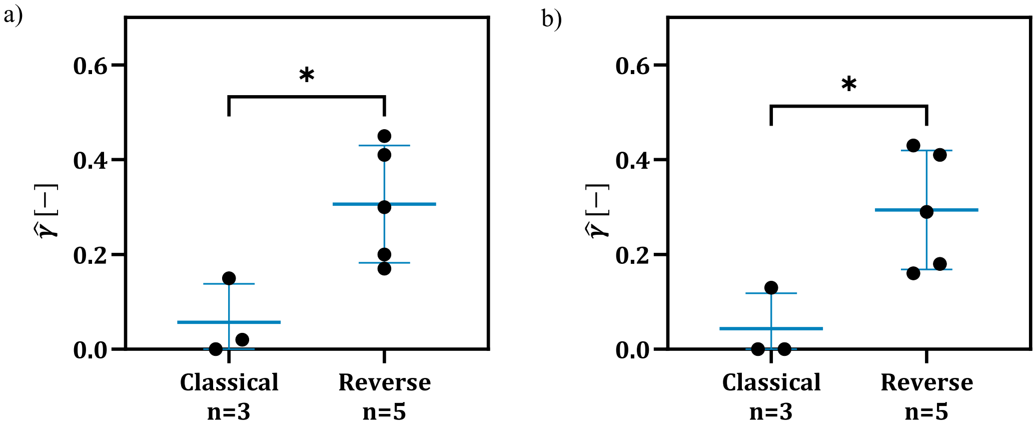

Supplemental Figure S5: Lingering frequency $\hat{\gamma}$ which yields the best fit to the previously reported distributions LDF from (1) classified by the resulting type of partitioning. The lingering frequency for the reverse partitioning group is statistically different from the classical partitioning group for both hematocrits: **(a)** $\overline{H}_{t,0} \approx 6.3\%$ ($H_r = 20\%$, $p = 3.6 \cdot 10^{-2}$) and **(b)** $\overline{H}_{t,0} \approx 8.7\%$ ($H_r = 30\%$, $p = 3.6 \cdot 10^{-2}$).

RBC migration across the separation surface

We analyzed the influence of LRBCs on the RBC distribution at the bifurcation. Similarly to the experiments at $\overline{H}_{t,0} \approx 5.2\%$ ($H_r = 10\%$), RBCs generally did not migrate across the separation surface (Table S2, "All RBC") even at the higher hematocrits. However, if only LRBCs are considered, we observed that the percentage of RBCs crossing the symmetry axis increases. The percentage of NLRBCs crossing the symmetry axis does not differ considerably from "All RBCs" (Table S2, "All NLRBC" vs. "All NLRBC"). For the subgroups of NLRBCs without any lingering event and NLRBCs during lingering event (LE), no major difference can be observed (Table S2 "NLRBC no LE" vs. "NLRBC during LE"). Overall, our data suggest that LRBCs do not have a direct influence on NLRBCs partitioning for the present configuration of our microfluidic network.

Supplemental Table S2: Statistics on all RBCs that were able to cross the symmetry axis while approaching the bifurcation. The RBC population was divided into subgroups: All RBC, LRBC, All NLRBC, NLRBC when there is no lingering event (LE) and NLRBC when there is a lingering event.

	$\overline{H}_{t,0} \approx 6.3\%$ [$H_r = 20\%$]	$\overline{H}_{t,0} \approx 8.7\%$ [$H_r = 30\%$]
**All RBC**	6.7%	8.1%
**LRBC**	36.0%	41.2%
**All NLRBC**	3.6%	4.3%
**NLRBC without LE**	4.3%	4.7%
**NLRBC during LE**	1.6%	3.6%

Velocity

Results reported in Table S3, Fig. S6a, Fig. S6c show that LRBCs are statistically significantly faster than NLRBCs at the inlet of the parent vessel for both hematocrits that we tested, but this difference disappears at the outlet. In the daughter vessels (Fig. S6b and Fig. S6d), the LRBCs have a statistically significantly lower velocity than the NLRBCs either at the inlet and at the outlet for both hematocrits.

Supplemental Table S3: Median velocity ($\tilde{u}$) in the parent vessel (0) and daughter vessels (1,2) for $\overline{H}_{t,0} \approx 6.3\%$ ($H_r = 20\%$) and $\overline{H}_{t,0} \approx 8.7\%$ ($H_r = 30\%$). A two-sample Kolmogorov–Smirnov test was performed to evaluate statistical differences between the u_{LRBC} and u_{NLRBC} distributions.

		$\overline{H}_{t,0} \approx 6.3\%$ [$H_r = 20\%$]				$\overline{H}_{t,0} \approx 8.7\%$ [$H_r = 30\%$]			
		In_0	Out_0	$In_{1,2}$	$Out_{1,2}$	In_0	Out_0	$In_{1,2}$	$Out_{1,2}$
$\mathbf{\tilde{u}_{LRBC}}$	$[mm/s]$	0.81	0.74	0.18	0.29	0.74	0.68	0.19	0.25
$\mathbf{\tilde{u}_{NLRBC}}$	$[mm/s]$	0.78	0.72	0.38	0.38	0.72	0.66	0.34	0.35
p-value		$3.8 \cdot 10^{-3}$	$1.4 \cdot 10^{-1}$	$3.5 \cdot 10^{-20}$	$1.1 \cdot 10^{-14}$	$5.1 \cdot 10^{-4}$	$1.0 \cdot 10^{-1}$	$1.9 \cdot 10^{-21}$	$3.9 \cdot 10^{-25}$

Circularity

Similar to what we found for $\overline{H}_{t,0} \approx 5.2\%$ ($H_r = 10\%$, cf. Supplementary Material, Additional analysis for experiment with $\overline{H}_{t,0} \approx 5.2\%$ [$H_r = 10\%$], Circularity), all RBCs in the parent vessel have a round shape for $\overline{H}_{t,0} \approx 6.3\%$ ($H_r = 20\%$) and $\overline{H}_{t,0} \approx 8.7\%$ ($H_r = 30\%$, Table S4, Fig. S7a, Fig. S7c). In the daughter vessels, the NLRBC eccentricity distribution is similar to what we found in the parent vessel, whereas LRBCs have a lower circularity (Fig. S7b and Fig. S7d).

Orientation

The RBC orientation from the experiments at $\overline{H}_{t,0} \approx 6.3\%$ ($H_r = 20\%$) and $\overline{H}_{t,0} \approx 8.7\%$ ($H_r = 30\%$) is broadly distributed in the parent vessel (Table S5, Fig. S8a and Fig. S8c) and it seems that the NLRBCs align more with the flow direction (similarly to $\overline{H}_{t,0} \approx 5.2\%$ [$H_r = 10\%$]). As explained in Supplementary Material, Additional analysis for experiment with $\overline{H}_{t,0} \approx 5.2\%$ ($H_r = 10\%$), Orientation section, this broad distribution may be related to the uncertainty of determining the orientation of an object with a discoid-like shape. In the daughter vessels, we observed quite the opposite situation where the LRBCs align with the flow direction and the NLRBCs do not seem to have a preferential orientation (Fig. S8b and Fig. S8d).

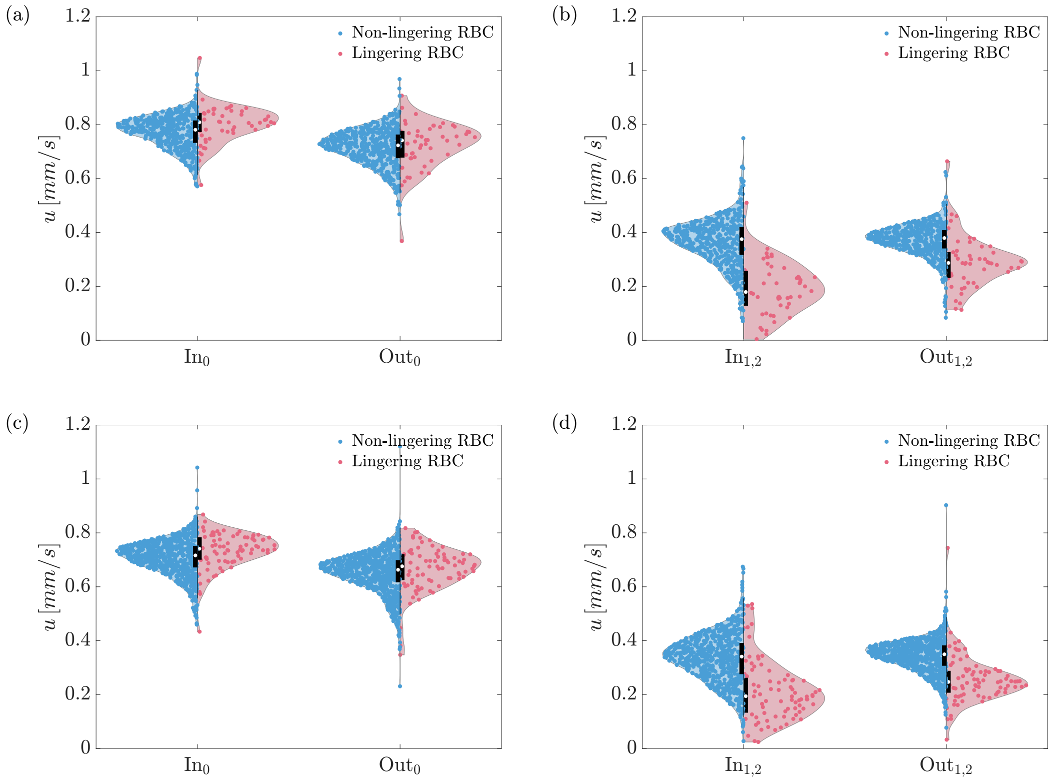

Supplemental Figure S6: RBC velocity distribution in the parent and daughter vessels for $\overline{H}_{t,0} \approx 6.3\%$ ($H_r = 20\%$) and $\overline{H}_{t,0} \approx 8.7\%$ ($H_r = 30\%$). **(a)** RBC velocity at the inlet (In_0) and outlet (Out_0) of the parent vessel for $\overline{H}_{t,0} \approx 6.3\%$ ($H_r = 20\%$). **(b)** RBC velocity at the inlet ($\mathrm{In}_{1,2}$) and outlet ($\mathrm{Out}_{1,2}$) of the daughter vessels for $\overline{H}_{t,0} \approx 6.3\%$ ($H_r = 20\%$). **(c)** RBC velocity at the inlet (In_0) and outlet (Out_0) of the parent vessel for $\overline{H}_{t,0} \approx 8.7\%$ ($H_r = 30\%$). **(d)** RBC velocity at the inlet ($\mathrm{In}_{1,2}$) and outlet ($\mathrm{Out}_{1,2}$) of the daughter vessels for $\overline{H}_{t,0} \approx 8.7\%$ ($H_r = 30\%$).

Supplemental Table S4: Median eccentricity ($\tilde{\epsilon}$) in the parent vessel (0) and daughter vessels (1,2) for $\overline{H}_{t,0} \approx 6.3\%$ ($H_r = 20\%$) and $\overline{H}_{t,0} \approx 8.7\%$ ($H_r = 30\%$). A two-sample Kolmogorov–Smirnov test was performed to evaluate statistical differences between the ϵ_{LRBC} and ϵ_{NLRBC} distributions.

	$\overline{H}_{t,0} \approx 6.3\%$ $[H_r = 20\%]$				$\overline{H}_{t,0} \approx 8.7\%$ $[H_r = 30\%]$			
	In_0	Out_0	$In_{1,2}$	$Out_{1,2}$	In_0	Out_0	$In_{1,2}$	$Out_{1,2}$
$\tilde{\epsilon}_{\mathbf{LRBC}}$	0.75	0.73	0.54	0.65	0.74	0.73	0.52	0.64
$\tilde{\epsilon}_{\mathbf{NLRBC}}$	0.75	0.75	0.76	0.76	0.75	0.75	0.74	0.74
p-value	$2.4{\cdot}10^{-1}$	$2.1{\cdot}10^{-2}$	$2.4{\cdot}10^{-24}$	$7.0{\cdot}10^{-15}$	$5.4{\cdot}10^{-1}$	$1.5{\cdot}10^{-1}$	$6.8{\cdot}10^{-31}$	$5.7{\cdot}10^{-19}$

Supplemental Table S5: Median orientation ($\tilde{\beta}$) in the parent vessel (0) and daughter vessels (1,2) for $\overline{H}_{t,0} \approx 6.3\%$ ($H_r = 20\%$) and $\overline{H}_{t,0} \approx 8.7\%$ ($H_r = 30\%$). $\beta = 0°$ indicates that an RBC is aligned with the centerline of the respective ROI (i.e. the RBC follows the main flow direction). A two-sample Kolmogorov–Smirnov test was performed to evaluate statistical differences between the β_{LRBC} and β_{NLRBC} distributions.

	$\overline{H}_{t,0} \approx 6.3\%$ $[H_r = 20\%]$				$\overline{H}_{t,0} \approx 8.7\%$ $[H_r = 30\%]$			
	In_0	Out_0	$In_{1,2}$	$Out_{1,2}$	In_0	Out_0	$In_{1,2}$	$Out_{1,2}$
$\tilde{\beta}_{\mathbf{LRBC}}$	8.5°	5.3°	11.1°	3.7°	1.9°	-1.2°	11.4°	4.0°
$\tilde{\beta}_{\mathbf{NLRBC}}$	5.7°	5.2°	4.2°	5.2°	3.3°	1.6°	1.1°	5.9°
p-value	$8.1{\cdot}10^{-2}$	$1.2{\cdot}10^{-2}$	$7.0{\cdot}10^{-8}$	$2.3{\cdot}10^{-2}$	$1.6{\cdot}10^{-1}$	$7\ .0{\cdot}10^{-2}$	$2.1{\cdot}10^{-13}$	$9.9{\cdot}10^{-4}$

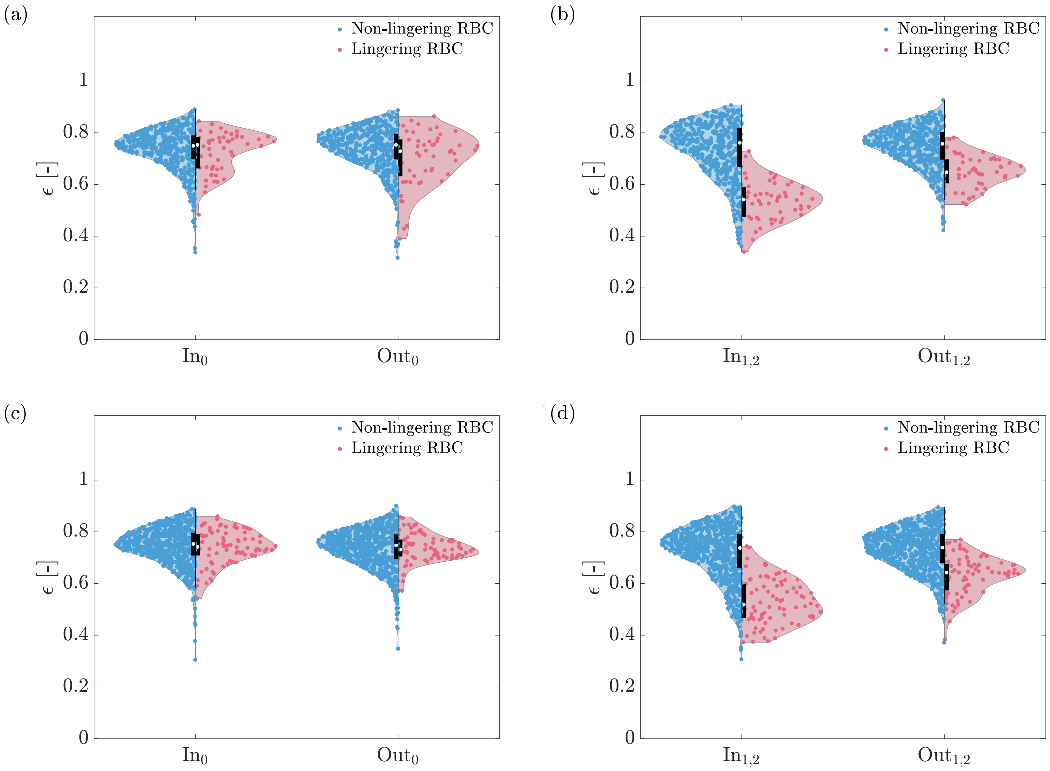

Supplemental Figure S7: RBC circularity (ϵ) distributions for $\overline{H}_{t,0} \approx 6.3\%$ ($H_r = 20\%$) and $\overline{H}_{t,0} \approx 8.7\%$ ($H_r = 30\%$), where $\epsilon = 1$ represents a perfect circle. **(a)** Circularity at the inlet (In_0) and outlet (Out_0) of the parent vessel $\overline{H}_{t,0} \approx 6.3\%$ ($H_r = 20\%$). **(b)** Circularity at the inlet ($\text{In}_{1,2}$) and outlet ($\text{Out}_{1,2}$) of the daughter vessels $\overline{H}_{t,0} \approx 6.3\%$ ($H_r = 20\%$). **(c)** Circularity at the inlet (In_0) and outlet (Out_0) of the parent vessel $\overline{H}_{t,0} \approx 8.7\%$ ($H_r = 30\%$). **(d)** Circularity at the inlet ($\text{In}_{1,2}$) and outlet ($\text{Out}_{1,2}$) of the daughter vessels $\overline{H}_{t,0} \approx 8.7\%$ ($H_r = 30\%$)

.

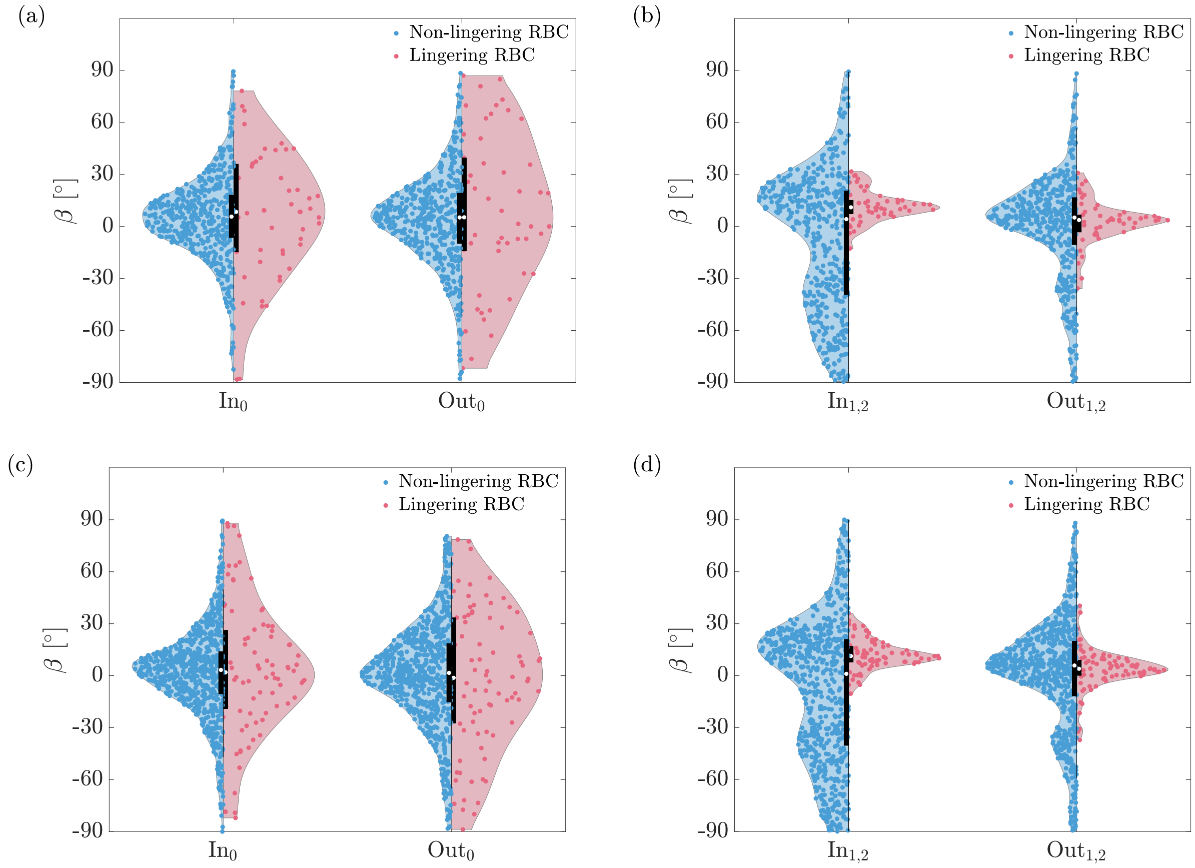

Supplemental Figure S8: RBC orientation (β) distributions for $\overline{H}_{t,0} \approx 6.3\%$ ($H_r = 20\%$) and $\overline{H}_{t,0} \approx 8.7\%$ ($H_r = 30\%$). $\beta = 0°$ indicates that an RBC is aligned with the centerline of the respective ROI (i.e. the RBC follows the main flow direction). **(a)** Orientation at the inlet (In_0) and outlet (Out_0) of the parent vessel for $\overline{H}_{t,0} \approx 6.3\%$ ($H_r = 20\%$). **(b)** Orientation at the inlet ($\mathrm{In}_{1,2}$) and outlet ($\mathrm{Out}_{1,2}$) of the daughter vessels for $\overline{H}_{t,0} \approx 6.3\%$ ($H_r = 20\%$). **(c)** Orientation at the inlet (In_0) and outlet (Out_0) of the parent vessel for $\overline{H}_{t,0} \approx 8.7\%$ ($H_r = 30\%$). **(d)** Orientation at the inlet ($\mathrm{In}_{1,2}$) and outlet ($\mathrm{Out}_{1,2}$) of the daughter vessels for $\overline{H}_{t,0} \approx 8.7\%$ ($H_r = 30\%$).

SUPPORTING REFERENCES

1. Mantegazza, A., F. Clavica, and D. Obrist, 2020. In vitro investigations of red blood cell phase separation in a complex microchannel network. *Biomicrofluidics* 14:014101. 10.1063/1.5127840.
